# Supplementary material for: Insight into the Natural History of Pathogenic Variant c.919-2A>G in the SLC26A4 Gene Involved in Hearing Loss: The Evidence for Its Common Origin in Southern Siberia (Russia)
Source: Genes (Basel). 2023 Apr 17;14(4):928. doi: 10.3390/genes14040928 (PMC10137394; doi:10.3390/genes14040928)
Supplement: Supplementary file 1 [file genes-14-00928-s001.zip › Supplementary Materials File S1.pdf]

Estimation of the c.919-2A>G age

The estimation of a mutation age is based on the expected decay of linkage disequilibrium between the mutation and alleles of surrounding genetic markers due to recombination (“genetic clock” concept). We applied two approaches for estimating the age of c.919-2A>G. The first (used when appropriate) was the single marker method based on intra-allelic variation of a single marker [37,65]. This approach implies analysis of alleles of the most distal markers which manifest significant linkage disequilibrium, while marker alleles with complete linkage disequilibrium (all mutant chromosomes carried the same allele) are considered to be uninformative. The estimation of the c.919-2A>G age by the single marker method was performed using following algorithm [37]:

$$g = \log[1 - Q / (1 - Pn)] / \log(1 - \Theta),$$

where g is the number of generations passed from the moment of the mutation appearance to the present; Q is the share of mutant chromosomes unlinked with the founder haplotype; Pn is the population frequency of allele included in the founder haplotype, and Θ is the recombinant fraction calculated from physical distance between marker and mutation (under the assumption that 1 cM = 1000 kb). In addition, to avoid possible underestimation of a mutation age, the Luria-Delbrűck correction, taking into account the demographic parameters, was applied [66,67]:

$$g_c = g + g_0, \quad g_0 = - (1/d) \ln(\Theta f_d),$$

where d is population growth rate, also assuming $f_d = e^d/(e^d-1)$ and $f_d \approx 1/d$ at small d values. The duration of one generation (g) was considered to be 25 years.

The second approach for estimating the age of c.919-2A>G was the DMLE+v2.3 software method (Disequilibrium Mapping and Likelihood Estimation, DMLE+v.2.3: http://dmle.org/) [36], which is based on multiply linked marker loci (the haplotype data) and uses the Markov Chain Monte Carlo algorithm for Bayesian estimation of the mutation age. For calculation of the mutation age by the DMLE+v2.3 software, the demographic parameters (population size, population growth rate and proportion of population sampled) are required in addition to the haplotype data and the map distances among marker loci and mutations. The parameter ‘proportion of population sampled’ for c.919-2A>G was calculated on the basis of our previous data [27]. The contemporary population size for Tuvinians, according to the Russian 2021 census, was 279789. Since the population growth rates for Tuvinian population could not be reliably estimated due to very limited knowledge of demographic variation of this population along its history, three plausible growth rates (0.05, 0.1 and 0.2) were used.
