# Supplementary material for: Insight into the Natural History of Pathogenic Variant c.919-2A>G in the SLC26A4 Gene Involved in Hearing Loss: The Evidence for Its Common Origin in Southern Siberia (Russia)
Source: Genes (Basel). 2023 Apr 17;14(4):928. doi: 10.3390/genes14040928 (PMC10137394; doi:10.3390/genes14040928)
Supplement: Supplementary file 1 [file genes-14-00928-s001.zip › Table S1.pdf]

Table S1. Allelic frequencies of analyzed SNPs according to data from the Genome Aggregation Database (gnomAD v3.1.2, https://gnomad.broadinstitute.org/, accessed on 13 February 2023).

	**SNPs Position (GRCh38.13), Alleles (distance from c.919-2A>G)**	**East Asian**	**South Asian**	**European (non-Finnish)**	**European (Finnish)**	**Ashkenazi Jewish**	**Middle Eastern**	**Amish**	**African / African American**	**Latino / Admixed American**	**Other**
1	**rs2248464** 7:107663177 A>T (20276)	0.6861	0.8506	0.7308	0.7630	0.7630	0.7911	0.6850	0.7991	0.7300	0.7646
2	**rs2248465** 7:107663183 C>T (20270)	0.4481	0.7933	0.7261	0.7630	0.6537	0.7500	0.6850	0.6651	0.7073	0.7157
3	**rs3801943** 7:107681221 T>A (2232)	0.1014	0.2271	0.2656	0.2949	0.2635	0.1013	0.1754	0.1120	0.2920	0.1925
4	**rs2712212 *** 7:107681270 A>G (2183)	0.1926	0.2219	0.2639	0.1467	0.2617	0.3312	0.4824	0.3011	0.2815	0.2965
	**c.919-2A>G** (7:107683453)										
5	**rs2395911 *** 7:107683668 G>T (215)	0.6376	0.5806	0.7437	0.6848	0.7336	0.6835	0.7796	0.8559	0.6652	0.7514
6	**rs2712211 *** 7:107685692 C>G (2024)	0.3580	0.4168	0.2553	0.3148	0.2666	0.3101	0.2204	0.07436	0.3253	0.2361
7	**rs3801940 *** 7:107685771 C>G (2318)	0.6320	0.4513	0.6230	0.6249	0.5530	0.4589	0.5890	0.6062	0.5516	0.5856
8	**rs2072064** 7:107694068 T>A (10615)	0.3566	0.4366	0.2545	0.3237	0.2380	0.2500	0.2165	0.04975	0.3101	0.2131
9	**rs2072065** 7:107694216 C>T (10763)	0.6313	0.3954	0.4478	0.4666	0.4741	0.3548	0.5022	0.4611	0.4497	0.4622

* – rs2712212, rs2395911, rs2712211, and rs3801940 were included for comparative analysis with the data from the study by Wu et al. [12].
