# Supplementary material for: Insight into the Natural History of Pathogenic Variant c.919-2A>G in the SLC26A4 Gene Involved in Hearing Loss: The Evidence for Its Common Origin in Southern Siberia (Russia)
Source: Genes (Basel). 2023 Apr 17;14(4):928. doi: 10.3390/genes14040928 (PMC10137394; doi:10.3390/genes14040928)
Supplement: Supplementary file 1 [file genes-14-00928-s001.zip › Table S2.pdf]

Table S2. Primer sequences for STR and SNP genotyping.

**STRs***	
D7S2420	(PET) F: 5’-CCTGTATGGAGGGCAAACTA-3’<br>R: 5’-AAATAATGACTGAGGCTCAAAACA-3’
D7S496	(VIC) F: 5’-AACAACAGTCAACCCACAAT-3’<br>R: 5’-GCTATAACCTCATAATAAACCAAAA-3’
D7S2459	(PET) F: 5’-AAGAAGTGCATTGAGACTCC-3’<br>R: 5’-CCGCCTTAGTAAAACCC-3’
D7S2456	(VIC) F: 5’-CTGGAAATTGACCTGAAACCTT-3’<br>R: 5’-ACAGGGGTCTCTCACACATATTA-3’
D7S525	(NED) F: 5’-GTTAGCCGAGATTGCCC-3’<br>R: 5’-CTTGCTGTTTAAGTACCACAAGTTC-3’
**SNPs**	
rs2248464<br>rs2248465	F: 5’-TCGTCCTCGCTTACCGCGTGT-3’<br>R: 5’-CCATCTGTCACAGACCTATGGTAGCT-3’
rs3801943<br>rs2712212	F: 5’-CCTCTGGGATAGGTAGGGCT-3’<br>R: 5’-AGTAAGGAAATGGGGGAG-3’
rs2395911	F: 5’-CATGGTTTTTCATGTGGGAAGATTC-3’<br>R: 5’-CAAATGGCTTGACGTTTATCTACACAC-3’
rs2712211<br>rs3801940	F: 5’-TAAGGCTGCTGCTCGTAGTT-3’<br>R: 5’-CTATAACAGAAGAGGTAAAA-3’
rs2072064<br>rs2072065	F: 5’-GAGCAGATATAGCATTTGATGAGATG-3’<br>R: 5’-CTCGGTGCGAGCCTTCCT-3’

* – Primer sequences for STRs genotyping were taken from the Ensembl Genome browser (http://www.ensembl.org). One (forward) from each primer pairs was marked on the 5′ end with the different fluorescent dyes.
