# Supplementary material for: Insight into the Natural History of Pathogenic Variant c.919-2A>G in the SLC26A4 Gene Involved in Hearing Loss: The Evidence for Its Common Origin in Southern Siberia (Russia)
Source: Genes (Basel). 2023 Apr 17;14(4):928. doi: 10.3390/genes14040928 (PMC10137394; doi:10.3390/genes14040928)
Supplement: Supplementary file 1 [file genes-14-00928-s001.zip › Table S3.pdf]

Table S3. The allelic frequencies of STRs (D7S2420, D7S496, D7S2459, D7S2456, and D7S525) in patients homozygous for c.919-2A>G and in the control sample.

STRs (distance to c.919-2A>G)	Allele*	Patients homozygous for c.919-2A>G (n=23)		Control sample (n=63)		*p*	δ
		Number of alleles	Frequency of alleles	Number of alleles	Frequency of alleles		
**D7S2420** (~ 0.43 Mb)	*276*	0	0.0	1	0.0079	0.7326	-0.0080
	278	46	1.0	10	0.0794	**$<10^{-31}$**	**1.0**
	280	0	0.0	17	0.1349	**0.0037**	-0.1560
	282	0	0.0	17	0.1349	**0.0037**	-0.1560
	284	0	0.0	24	0.1905	**$<10^{-3}$**	-0.2353
	286	0	0.0	19	0.1508	**0.0018**	-0.1776
	288	0	0.0	21	0.1667	**$<10^{-3}$**	-0.2000
	290	0	0.0	8	0.0635	0.0780	-0.0678
	292	0	0.0	6	0.0476	0.1496	-0.0500
	294	0	0.0	3	0.0238	0.3906	-0.0244
	Total	46	1.0	126	1.0		
**D7S496** (~ 0.17 Mb)	*118*	0	0.0	23	0.1825	**$<10^{-3}$**	-0.2233
	120	46	1.0	3	0.0238	**$<10^{-37}$**	**1.0**
	126	0	0.0	1	0.0079	0.7326	-0.0080
	128	0	0.0	4	0.0317	0.2843	-0.0328
	130	0	0.0	14	0.1111	**0.0104**	-0.1250
	132	0	0.0	13	0.1032	**0.0147**	-0.1150
	134	0	0.0	61	0.4841	**$<10^{-10}$**	-0.9385
	136	0	0.0	3	0.0238	0.3906	-0.0244
	138	0	0.0	3	0.0238	0.3906	-0.0244
	144	0	0.0	1	0.0079	0.7326	-0.0080
	Total	46	1.0	126	1.0		
			**c.919-2A>G**				
**D7S2459** (~ 7.6 kb)	*139*	0	0.0	5	0.0397	0.2064	-0.0413
	141	0	0.0	10	0.0794	**0.0403**	-0.0862
	143	0	0.0	3	0.0238	0.3906	-0.0244
	145	0	0.0	59	0.4683	**$<10^{-10}$**	-0.8806
	147	46	1.0	27	0.2143	**$<10^{-22}$**	**1.0**
	149	0	0.0	18	0.1429	**0.0026**	-0.1667
	151	0	0.0	4	0.0317	0.2843	-0.0328
	Total	46	1.0	126	1.0		
**D7S2456** (~ 0.36 Mb)	*244*	46	1.0	73	0.5794	1.0	**1.0**
	246	0	0.0	36	0.2857	**$<10^{-5}$**	-0.4000
	248	0	0.0	2	0.0159	0.5355	-0.0161
	250	0	0.0	14	0.1111	**0.0104**	-0.1250
	252	0	0.0	1	0.0079	0.7326	-0.0080
	Total	46	0.0	126	1.0		
**D7S525** (~ 2.32 Mb)	*209*	0	0.0	2	0.0159	0.5355	-0.0161
	219	0	0.0	2	0.0159	0.5355	-0.0161
	221	1	0.0217	36	0.2857	**$<10^{-4}$**	-0.3696
	223	0	0.0	5	0.0397	0.2064	-0.0413
	225	1	0.0217	4	0.0317	0.5956	-0.0103
	227	42	0.9130	50	0.3968	**$<10^{-9}$**	**0.8558**
	229	2	0.0435	19	0.1508	**0.0430**	-0.1264
	231	0	0.0000	8	0.0635	0.0780	-0.0678
	Total	46	1.0	126	1.0		

* – Designation of the STR allele corresponds to its size in nucleotides; the maximum indices of linkage disequilibrium (δ) and statistically significant ($p < 0.05$) differences in allele frequencies between the c.919-2A>G homozygotes and the control sample are in bold.
