# Supplementary material for: Insight into the Natural History of Pathogenic Variant c.919-2A>G in the SLC26A4 Gene Involved in Hearing Loss: The Evidence for Its Common Origin in Southern Siberia (Russia)
Source: Genes (Basel). 2023 Apr 17;14(4):928. doi: 10.3390/genes14040928 (PMC10137394; doi:10.3390/genes14040928)
Supplement: Supplementary file 1 [file genes-14-00928-s001.zip › Table S4.pdf]

Table S4. The genotypes and the allelic frequencies of analyzed SNPs in patients homozygous for c.919-2A>G and in the control sample.

SNPs (distance from c.919-2A>G)	Genotypes	Homozygotes for c.919-2A>G (n=23)	Controls (n=23)	Alleles	Homozygotes for c.919-2A>G		Controls		*p*	δ
					Number of alleles	Frequency of alleles	Number of alleles	Frequency of alleles		
**rs2248464** (20276)	**AA**	23	4	**A**	46	1.0	26	0.2063	**0.1697×10^{-22}**	**1.0**
	**AT**	0	18							
	**TT**	0	41	**T**	0	0.0	100	0.7937	**0.1687×10^{-22}**	-3.8462
	**Total**	23	63							
**rs2248465** (20270)	**CC**	23	9	**C**	46	1.0	40	0.3175	**0.3332×10^{-17}**	**1.0**
	**CT**	0	22							
	**TT**	0	32	**T**	0	0.0	86	0.6825	**0.3300×10^{-17}**	-2.1500
	**Total**	23	63							
**rs3801943** (2232)	**TT**	23	45	**T**	46	1.0	107	0.8492	1.0	**1.0**
	**TA**	0	17							
	**AA**	0	1	**A**	0	0.0	19	0.1508	**0.1818×10^{-2}**	-0.1776
	**Total**	23	63							
**rs2712212 *** (2183)	**AA**	23	28	**A**	46	1.0	84	0.6667	1.0	**1.0**
	**AG**	0	28							
	**GG**	0	7	**G**	0	0.0	42	0.3333	**0.2168×10^{-6}**	-0.5000
	**Total**	23	63							
				**c.919-2A>G**						
**rs2395911 *** (215)	**GG**	23	9	**G**	46	1.0	44	0.3492	**0.6282×10^{-16}**	**1.0**
	**GT**	0	26							
	**TT**	0	28	**T**	0	0.0	82	0.6508	**0.6211×10^{-16}**	-1.8636
	**Total**	23	63							
**rs2712211 *** (2024)	**CC**	0	26	**C**	0	0.0	78	0.6190	**0.9734×10^{-15}**	-1.6250
	**CG**	0	26							
	**GG**	23	11	**G**	46	1.0	48	0.3810	1.0	**1.0**
	**Total**	23	63							
**rs3801940 *** (2318)	**CC**	23	14	**C**	46	1.0	56	0.4444	1.0	**1.0**
	**CG**	0	28							
	**GG**	0	21	**G**	0	0.0	70	0.5556	**0.1503×10^{-12}**	-1.2500
	**Total**	23	63							
**rs2072064** (10615)	**TT**	0	25	**T**	0	0.0	75	0.5952	**0.6892×10^{-14}**	1.4706
	**TA**	0	25							
	**AA**	23	13	**A**	46	1.0	51	0.4048	1.0	**1.0**
	**Total**	23	63							
**rs2072065** (10763)	**CC**	23	16	**C**	46	1.0	64	0.5079	1.0	**1.0**
	**CT**	0	32							
	**TT**	0	15	**T**	0	0.0	62	0.4921	**0.1391×10^{-10}**	-0.9688
	**Total**	23	63							

* – rs2712212, rs2395911, rs2712211, and rs3801940 were included for comparative analysis with the data from the study by Wu et al. [12]. The maximum indices of linkage disequilibrium (δ) and statistically significant ($p < 0.05$) differences in allele frequencies between the c.919-2A>G homozygotes and the control sample are in bold.
